# Concurrent activation potentiation improves lower-limb maximal strength but not dynamic balance control in rugby players

**DOI:** 10.3389/fbioe.2023.1270322

**Published:** 2024-01-05

**Authors:** Alex Rizzato, Vittorio G. Dalla Costa, Matteo Bozzato, Antonio Paoli, Giuseppe Marcolin

**Affiliations:** ^1^ Department of Biomedical Sciences, University of Padova, Padua, Italy; ^2^ School of Human Movement Sciences, University of Padova, Padua, Italy

**Keywords:** postural control, center of pressure, external perturbations, leg extension, isometric strength, rate of force development

## Abstract

Concurrent activation potentiation (CAP) increases athletic performance by activating muscles not involved in the performed activity. Among the CAP strategies, jaw clenching is the most practical to implement in sports contexts. Muscle strength and balance control are essential among rugby players to cope tackles. Besides combat sports, mouthguard has become mandatory also in rugby. Therefore, this study aimed to understand whether mouthguard jaw-clenching could improve rugby players’ dynamic balance and quadriceps isometric strength. Thirteen rugby players were tested under maximal-bite (MB) and no-bite (NB) conditions. During standing balance tests, an electro-actuated platform with a force plate screwed on it allowed for the perturbation of the support base of the rugby players. A verbal signal warned the subject that the perturbation was coming, mentally recalling an in-field expected collision. In the first 2.5 s window after the perturbation, the center of pressure (CoP) displacement and mean velocity were measured. The first peak, the maximal oscillations, and the standard deviation of the anterior-posterior CoP trajectory were calculated within the same time window. In the isometric leg-extension test, a custom-built chair instrumented with a uni-axial load cell allowed to collect the maximal strength and rate of force development (RFD). Mouthguard jaw-clenching did not affect CoP-related parameters but increased maximal strength (*p* < 0.05) and RFD (0–50 ms: *p* < 0.01; 50–100 ms: *p* < 0.001; 100–150 ms: *p* < 0.05) in the isometric leg-extension test. Mouthguard jaw-clenching alone could be useful to increase lower-limb maximal isometric strength and RFD but did not improve dynamic balance performance in a sport-oriented postural balance test.

## Introduction

Concurrent activation potentiation (CAP) was first defined as the phenomenon that leads to acutely increase muscular and athletic performance, achieved by simultaneously activating muscles not directly involved in the activity (Ebben, 2006). This synchronous activation of muscles not involved in the primary performance was named remote voluntary contraction (RVC). RVC strategies range from single RVC, where only one muscle group is remotely activated, to combined RVCs, where different muscle groups are called into play concurrently (Allen et al., 2018). Several examples of RVCs have been studied, including hand gripping (Ebben et al., 2008b), jaw clenching (Ebben et al., 2008a; Mace and Allen, 2020), and the Valsalva maneuver (Ebben, 2006; Ebben et al., 2010b). For instance, Ebben and colleagues showed that RVC strategies (i.e., jaw clenching, hand gripping, and the Valsalva maneuver) produced a higher peak torque and power output during a concentric isokinetic knee extension compared to no-RVC condition (Ebben et al., 2010b). Moreover, the greater the amount and intensity of concomitant RVCs, the greater the increase in performance during strength tasks. In detail, concomitant RVCs (i.e., hand gripping, jaw clenching, and Valsalva maneuver) during a maximal isometric leg extension task produced greater strength values than each RVC alone (Ebben et al., 2008b). Among the RVCs previously investigated, jaw clenching is the most applicable and practical to implement in sports contexts.

Besides combat sports such as karate and boxing, mouthguard use has become widespread and mandatory in team sports such as rugby. Findings provided evidence that mouthguard use is a simple and effective injury prevention strategy for rugby players (Quarrie et al., 2005). Although mouthguard use was always intended for self-protection (Yamada et al., 1998), recent studies showed a performance increment related to jaw-clenching, opening new insights into athletic success. Allen and colleagues found improvements in peak force, normalized peak force, and rate of force development (RFD) during the isometric clean pull assessment when subjects maximally clenched their jaw, regardless of mouthpiece condition (Allen et al., 2018).

If the acute effects of jaw-clenching-induced CAP on strength and power have been exhaustively studied (Ebben et al., 2008b; Issurin and Verbitsky, 2013; Mace and Allen, 2020), there are other physiological capacities related to sports performance that could be potentially affected by CAP. The dynamic balance represents one of these in team sports with multiple contacts. Indeed, rugby players regularly experience impacts associated with falls, collisions, tackles, rucks, and scrums when playing or training (Hume et al., 2017). Hence, better dynamic balance control could be associated with enhanced athletic performance and reduced sports injuries (Hrysomallis, 2007; Han et al., 2015; Mirmoezzi and Taheri, 2018).

Nonetheless, although a few studies showed no influence of jaw clenching on postural balance performance (Dunn-Lewis et al., 2012; Golem and Arent, 2015), results are not entirely exhaustive on this issue because of some lacking methodologies. Both the studies above (Dunn-Lewis et al., 2012; Golem and Arent, 2015) assessed dynamic balance only in the mediolateral direction and did not state whether jaw clenching was maximal or submaximal. Recently, Nam and colleagues revealed that using a customized mouthguard in professional basketball players did not acutely improve dynamic balance performance (Nam et al., 2020). Dynamic balance ability was measured with a one-legged standing test assessing the left and right postural sway through an unstable device (i.e., Posturomed 202, Pullenereuth, Germany). However, the authors should have stated what the dynamic balance test precisely consists of (i.e., amplitudes, direction, and frequency of the oscillations). Moreover, the application of a sport-oriented postural balance test should be recommended for the assessment of athletes (Marcolin et al., 2019).

Therefore, considering the limitations of the previous studies (Dunn-Lewis et al., 2012; Golem and Arent, 2015; Nam et al., 2020), the role of mouthguard jaw clenching on postural balance performance in sports deserves further exploration. Indeed, no studies investigated whether mouthguard jaw clenching could acutely improve dynamic balance performance in a sport-oriented test. Therefore, since rugby players require high levels of postural balance and strength to face several tackling events during the game and are well familiar with wearing a mouthguard, the aim of the present study was twofold: 1) to understand whether jaw-clenching-induced CAP could improve the dynamic balance performance throughout external perturbation conditions in rugby players; 2) to investigate whether jaw-clenching-induced CAP could improve lower-limb isometric strength and rate of force development.

## Methods

### Participants

After an email advertisement to local rugby teams, thirteen competitive rugby players volunteered for the study (all males; mean ± standard deviation (SD): 23 ± 1.83 years; 83.31 ± 11.12 kg; 1.78 ± 0.08 m). Before enrollment, all the subjects were screened through a telephone interview, and all were eligible for the study. Inclusion criteria for participants’ recruitment were: i) current competitive rugby practice, ii) aged between 20 and 30 years, and iii) competitive rugby practice in at least the previous 5 years. Moreover, the following exclusion criteria were considered: no history of i) orthopedic injuries in the last 3 months, ii) concussions or neurological diseases, and iii) sight, hearing, or vestibular disorders.

### Experimental design

**FIGURE 1:**
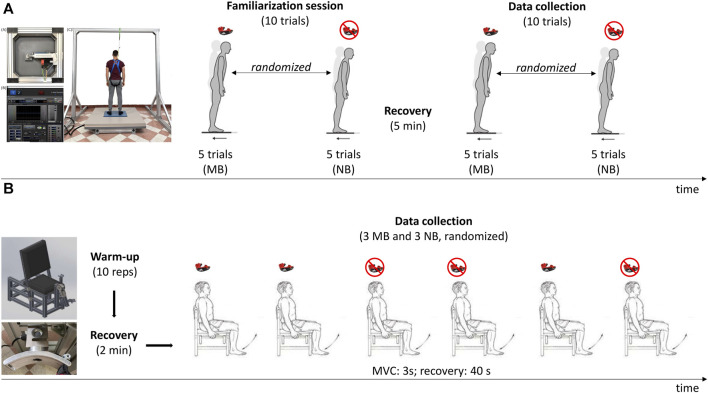
Experimental design of the dynamic balance tests over the electro-actuated movable plate **(A)** and strength assessment on the custom-built chair **(B)**. MB: maximal-bite; NB: no-bite; MVC: maximal voluntary contraction.

The experimental protocol received approval from the Human Ethical Committee of the Department of Biomedical Science of the University of Padova (protocol code: HEC-DSB/07-21) and adhered to the principles of the Declaration of Helsinki. After being informed about all methods and procedures adopted during the experimental protocol, participants gave their written consent but were free to renounce the study at any time. In detail, the experimental cross-sectional design evaluated dynamic balance control (Figure 1A) and quadriceps maximal isometric strength (Figure 1B). During the trials, subjects wore an over-the-counter boil-and-bite mouthguard. The subjects randomly underwent maximal-bite (MB) and no-bite (NB) conditions for dynamic balance and maximal isometric strength tests.

### Dynamic balance control

Dynamic balance control (Figure 1A) was assessed with a servo-controlled electrically driven movable platform (EnginLAB s. r.l., Italy), already described elsewhere (Rizzato et al., 2023). The system consists of an electro-actuated cylinder connected to a 135 cm × 135 cm plate, with linear motion allowed by two ball-type linear guideways. A 60 cm × 40 cm dynamometric platform (AMTI BP400600, United States) was placed over the 135 cm × 135 cm movable plate to calculate the CoP trajectory during the perturbations. The dynamometric platform had the following characteristics: sampling frequency of 200 Hz; average CoP accuracy less than 0.2 mm; crosstalk values typically ±0.05% of the applied load; measurement accuracy typically ±0.1% of the applied load. The signal was recorded with the software Balance Clinic 1.4.2. An external trigger synchronized the dynamometric and servo-controlled electrically driven movable platforms. The displacement of the movable platform was set to 100 mm, the ramp rate was 400 mm/s, and the direction of the motion was backward with respect to the standing position of the subject. Each trial lasted 20 s.

During the testing session, subjects were instructed to stand barefoot with extended legs, place arms along their sides, and gaze at a vertical line on a white wall in front of them. The feet position on the dynamometric platform was standardized equal to the shoulder width and marked using tape. The trial was considered invalid if the subject made a step consequent to the perturbation. Then, the feet were repositioned in the marked position before moving on to the successive trials. All participants wore a safety harness attached to an overhead frame to prevent falling in case of loss of postural balance due to platform shifting. The subjects wore a customized over-the-counter mouthguard during the whole testing session. For each trial, the operator anticipated the movement of the platform shouting “Hop!”. The verbal signal warned the subject that the perturbation of the base of support was coming. This procedure aimed to test the dynamic balance after perturbations in expected conditions, like those in the rugby field where the player performs or receives a head-on tackle consciously. Subjects were required to respond to the perturbation with the greatest stability possible. In the MB condition, subjects were instructed to bite maximally on the mouthguard at the verbal signal and keep biting until the platform stopped. In the NB, subjects wore the mouthguard but were not requested to bite at the verbal signal. Ten trials were performed in MB and NB conditions with a 30-s recovery between trials. The first five trials of each condition were administered to familiarize with the setup and the procedure, and thus, they were not included in the data analysis.

### Maximal isometric strength

The dominant lower limb strength was evaluated through an isometric maximal voluntary contraction (MVC) of the quadriceps. The experimental setup consisted of a custom-built chair (Figure 1B) instrumented with a uni-axial load cell (MuscleLab - Ergotest Technology) positioned 3 cm above the malleolus. The subjects performed the MVC seated with the knee flexed at 90° and secured to the chair with straps to minimize additional body movements. Subjects were asked to keep their hands crossed over the chest for the whole test duration. Before the test, ten submaximal warm-up contractions were performed. After the warm-up, three maximal trials, both in MB and NB conditions, were randomly performed with 40 s of recovery in between. The duration of each MVC was 3 s, during which the operator verbally prompted the subject (Andreacci et al., 2002).

### Data analysis

**FIGURE 2:**
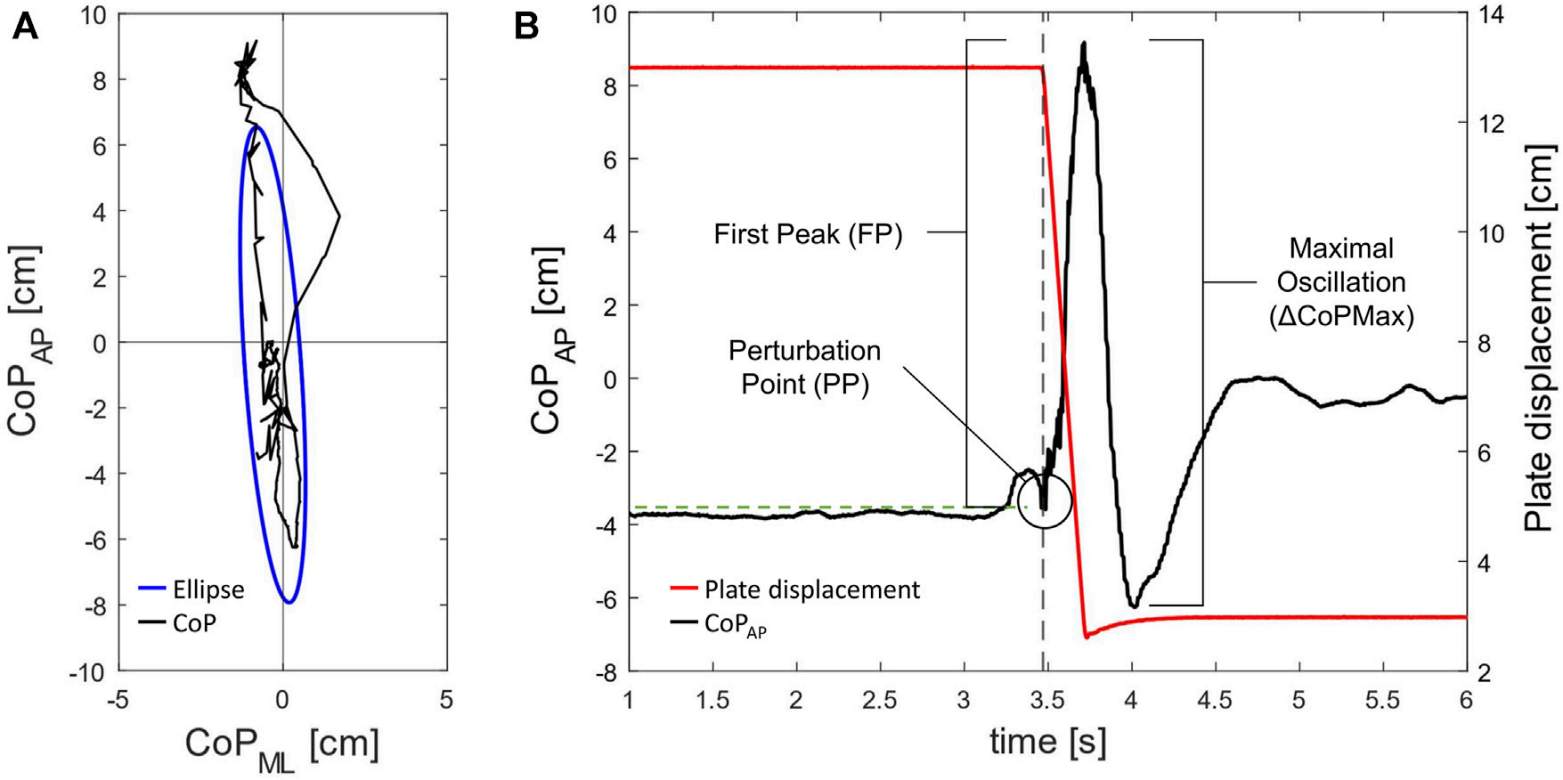
Center of pressure (CoP) trajectory (in black) and 95th percentile ellipse (in blue) within the 2.5 s time window **(A)**. First peak (FP) and maximal oscillation (ΔCoPMax) referred to the anterior-posterior CoP trajectory **(B)**; the grey dotted line marks the perturbation point (PP), and the green dotted line marks the mean value of the CoP trajectory before the PP.

As previously presented elsewhere (Rizzato et al., 2023), we calculated a set of CoP-related parameters over a 2.5-s time window from the perturbation point (PP), representing the instant the platform started moving. Area95 is the area of the 95th percentile ellipse measured in cm^2^, and Unit Path is the average CoP velocity in cm∙s^−1^. Moreover, we calculated three additional parameters (Figure 2) to deepen the postural responses in the direction of the perturbation (i.e., anterior-posterior): the first peak (FP), the maximal oscillations (ΔCoPMax), and the post-perturbation variability (PPV). The FP represents the difference between the maximal peak reached by the CoP displacement after the PP and the mean value of the anterior-posterior CoP displacement before the PP. The ΔCoPMax represents the sum of the absolute values of FP and the subsequent peak. Then, PPV is defined as the SD of the CoP anterior-posterior displacement over the 2.5-s time window to quantify the variability of the CoP displacement. The PPV is an index of the efficiency of the subject in controlling the body oscillations immediately after the external perturbation to reach a new quiet condition. In each condition, the CoP parameters were averaged among the five trials. Finally, the highest lower-limb peak force (Fmax) in both MB and NB conditions was considered among the three trials and expressed in Newtons. Moreover, the rate of force development (RFD) was calculated over the following time windows from the onset of force production: 0–50 ms (RFD_0-50_), 50–100 ms (RFD_50-100_), and 100–150 ms (RFD_100-150_).

### Statistical analysis

The *a priori* power analysis calculation (G*Power 3.1.9.2, Heinrich Heine University, Düsseldorf, Germany) showed that a sample size of 12 participants and a high effect size of 0.8 would provide a statistical Power of 0.8. The Shapiro-Wilk test was used to check the data normality distribution. Moreover, a paired *t*-test was employed to assess any significant difference between variables in MB and NB conditions for both dynamic balance (i.e., Area95, Unit Path, First Peak, ΔCoPMax, and PPV) and maximal isometric strength (Fmax, RFD_0-50_, RFD_50–100_, and RFD_100–150_) parameters. Data were processed with the software package JASP for Windows (Version 0.16.2.0) and presented as mean ± SD. The significant level for differences was set to *p* < 0.05.

## Results

**FIGURE 3:**
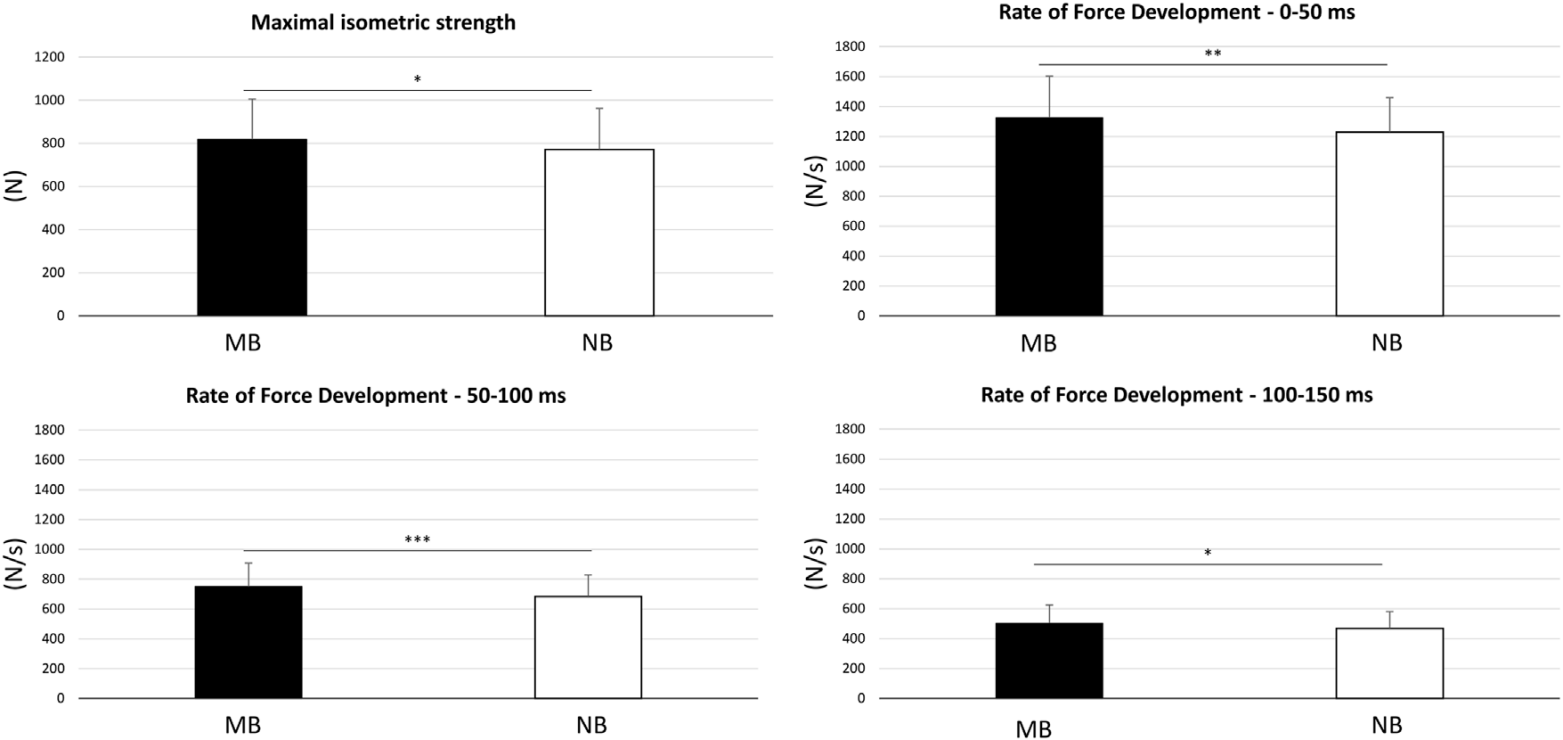
Results of the lower-limb maximal isometric strength and rate of force development within 0–50 ms, 50–100 ms, and 100–150 ms time windows. Data are presented as mean ± standard deviation. MB: maximal-bite; NB: no-bite; * (*p* < 0.05); ** (*p* < 0.01); *** (*p* < 0.001).

All subjects (n = 13) regularly completed the trials and were included in the data analysis. Table 1 summarizes the dynamic balance results with no statistically significant differences between the MB and NB conditions detected. Figure 3 shows the maximal isometric strength results. The paired-sample *t*-test showed significantly higher (*p* < 0.05) values of Fmax in the MB (817.81 ± 186.94 N) than NB (770.66 ± 190.43 N) condition. Similarly, all the RFD parameters resulted significantly higher in the MB condition (i.e., RFD_0–50_: 1324.19 ± 276.94 N/s; RFD_50–100_: 749.76 ± 158.31 N/s; RFD_100–150_: 502.15 ± 122.40 N/s) compared to NB (i.e., RFD_0-50_: 1228.06 ± 230.28 N/s; RFD_50–100_: 638.54 ± 144.84 N/s; RFD_100-150_: 467.33 ± 114.14 N/s) condition.

**TABLE 1:** Results of balance parameters in the maximal-bite (MB) and no-bite (NB) conditions. Data are presented as mean ± standard deviation. ΔCoPMax: maximal oscillations; PPV: post-perturbation variability.

	MB	NB	*p*-value
Area95 (cm^2^)	28.71 ± 8.47	28.24 ± 10.08	0.86
Unit Path (cm∙s^−1^)	27.23 ± 2.63	27.06 ± 3.50	0.71
First Peak (cm)	11.39 ± 1.25	11.13 ± 2.02	0.49
ΔCoPMax (cm)	13.25 ± 1.07	12.58 ± 2.13	0.16
PPV	3.11 ± 0.61	2.98 ± 0.62	0.39

## Discussion

This study aimed to understand whether jaw-clenching-induced CAP could improve the dynamic balance under external perturbation and strength in rugby players. Our research provided the first CoP-related evidence that jaw-clenching-induced CAP did not improve dynamic balance performance in rugby players. Even though, on one side, jaw clenching was shown to improve reflex facilitation (Takada et al., 2000) and static balance (Ringhof et al., 2015), on the other side, maximal clenching did not show any influence on dynamic stability (Dunn-Lewis et al., 2012; Nam et al., 2020). However, some methodological choices of these past studies could have affected the strength of their findings. Specifically, Golem et al. assessed dynamic balance scoring the amount of time the subjects could stay over a pivoting board in a central position with a mediolateral range of error of 5°(Golem and Arent, 2015), thus without considering CoP-related parameters that are the most employed for postural balance assessment (Paillard and Noé, 2015). Moreover, Dunn-Lewis and colleagues examined trained subjects wearing a customized mouthguard and performing different physical tests. In a 20-s postural balance test, the subjects stood on their non-dominant leg on top of a force plate, but only the medio-lateral oscillations were measured. Moreover, although these authors found no improvement in dynamic balance (Dunn-Lewis et al., 2012; Golem and Arent, 2015), they failed to describe the intensity of jaw clenching.

Similar to a previous study (Ringhof et al., 2016), our methodological approach encompasses balance control, strength production, and reaction to a perturbed event. Ringhof and colleagues showed that sub-maximal biting did not influence dynamic stability in recovery from a simulated forward fall by taking a single step (Ringhof et al., 2016). However, despite the authors analyzed force-plate-derived parameters, the simulated fall drove this setting away from a proper ecological approach. In this regard, since postural strategies are influenced by the specific motor skills and environment of a sport (Paillard, 2014), our sport-oriented test, even though far from actual on-field conditions, called into play a tackle-induced unbalance in a laboratory environment. Specifically, the verbal warning of the experimenter intended to mentally recall the in-field situation where a player is aware of receiving a head-on tackle.

Our findings based on the CoP-related parameters showed that CAP has no positive effect on dynamic balance performance. Although these parameters represent a novelty in this field, they mostly assess the efficacy of the earliest feet-in-place postural responses to the perturbation. These postural responses depend on the spinal cord-mediated reflexes with the shortest latencies. Thus, reflex postural responses could explain why jaw-clenching did not positively influence dynamic balance performance in our study.

The second aim of the study was to investigate whether the mouthguard could influence maximal lower-limb strength and rate of force development. Our results demonstrated that maximal jaw-clenching on the mouthguard improved rugby players’ isometric maximal strength. These results are in line with the previous literature where CAP acted as a performance enhancer in several acute strength and power tasks (Ebben, 2006; Ebben et al., 2008a; Ebben et al., 2010b). To the best of our knowledge, while the mouthguard as a self-protection device has been widely studied in rugby (Blignaut et al., 1987; Chalmers, 1998), only two studies investigated its sport-oriented role in a rugby players’ cohort (Duarte-Pereira et al., 2008; Dias et al., 2022). Results suggested that maximally clenching the mouthguard enhanced players’ height in the countermovement jump test (Duarte-Pereira et al., 2008) and force and acceleration peaks in the ballistic bench press exercise (Dias et al., 2022). Thus, our study first provided an increment of the lower-limb peak strength associated with mouthguard use in rugby players in an isometric leg extension task. Moreover, a recent review confirmed that jaw clenching while wearing custom-made, bite-aligning oral devices might benefit lower limb strength and power, especially in jumping ability and knee extension movements (Miró et al., 2021). Several hypotheses on the mechanisms associated with an RVC-induced strength increase have been proposed. The first calls into play the intercortical connections between the different motor areas of the brain. Hence, when the motor cortex is activated from the jaw clenching, other brain areas send impulses to the muscles, prime movers, of the performed action (e.g., leg extension) (Ebben, 2006). A second hypothesis regards the enhanced excitability of spinal motor neurons. Indeed, jaw clenching could increase the activity of α motor neurons, γ loops, and muscle spindles strengthened by the cortical afferent input (Ebben, 2006). A last explanation was given by the increased excitability of the Hoffman reflex following the afferent input from the oral-facial region activity (Sugawara et al., 2005).

Our results on RFD increment during the first 150 ms in the maximal-bite condition are consistent with previous investigations of the effects of CAP on muscular performance. Indeed, Ebben et al. found an average improvement (19.5%) in the RFD during a countermovement jump test while the subjects clenched their jaw maximally compared to the non-clenching condition (Ebben et al., 2008a). Later, the same authors demonstrated a significant increase in the RFD during the first 100 ms while performing both the back squat and jump squat exercise in the CAP condition compared with the no-CAP condition (Ebben et al., 2010a). Thus, the improvement of RFD in the isometric test added to the previous evidence that jaw clenching increased power during explosive dynamic strength exercises. Moreover, further investigations could deepen the role of jaw clenching in a possible relationship between the well-known improvements in muscular performance indicators (i.e., strength and power) and the reactive postural control (i.e., taking a step following a perturbation event).

The present study has some potential limitations to acknowledge. First, although the external perturbation aimed to simulate a destabilization consequent to a head-on tackle, our methodology did not fully reflect in-game collisions. Indeed, the ramp rate chosen in our experimental protocol (i.e., 400 mm/s) was lower compared to real-game tackles (Hendricks et al., 2012), but it was the maximal magnitude that would have prevented the athletes from taking a step. Secondly, although the tool used and the sport-oriented approach to test dynamic balance represented a novelty in this field, the small sample size could limit the generalizability of the findings to a larger population.

In conclusion, our study provided the first CoP-related evidence that jaw-clenching-induced CAP did not improve dynamic balance performance during an external perturbation of the base of support in a sport-oriented postural balance test. Since maximal jaw clenching improved the peak force and RFD in the isometric lower-limb task, we encourage rugby athletes to bite the mouthguard to improve the efficacy of their strength and power tasks. Moreover, since strength and power may be considered contributory physiologic attributes to postural balance, the relationship between jaw clenching and dynamic balance deserves further investigation.

## Data Availability

The raw data supporting the conclusion of this article will be made available by the authors, without undue reservation.
